# Sleep Duration and Risk of Lung Cancer in the Physicians' Health Study

**DOI:** 10.3779/j.issn.1009-3419.2014.09.02

**Published:** 2014-09-20

**Authors:** Owais KHAWAJA, Andrew B. PETRONE, Sohaib ALEEM, Kamran MANZOOR, John M GAZIANO, Luc DJOUSSE

**Affiliations:** 1 Section of Pulmonary and Critical Care Medicine, Dartmouth Hitchcock Medical Center, Lebanon, USA; 2 Divisions of Aging, Brigham and Women's Hospital and Harvard Medical School, Boston, USA; 3 Section of General Internal Medicine, Dartmouth Hitchcock Medical Center, Lebanon, USA; 4 Massachusets Veterans Epidemiology and Research Information Center (MAVERIC), Boston Veterans Affairs Healthcare System, Boston, USA; 5 Preventive Medicine, Brigham and Women's Hospital and Harvard Medical School, Boston, USA; 6 Geriatric Research, Education, and Clinical Center (GRECC), Boston Veterans Affairs Healthcare System, Boston, USA

**Keywords:** Sleep, Lung neoplasms, Risk factors, Epidemiology

## Abstract

**Background and Objectives:**

Lung cancer is the most common cancer and cancer related cause of death worldwide. However, the association between sleep duration and incident lung cancer has not been investigated in a prospective cohort study.

**Methods:**

We prospectively examined the association between sleep duration and incident lung cancer in a cohort of 21, 026 United States (US) male physicians. Self-reported sleep duration was ascertained during 2002 annual follow-up questionnaire. Incident lung cancer was ascertained through yearly follow-up questionnaires. Cox regression was used to estimate relative risk of incident lung cancer.

**Results:**

The average age at baseline was 68.3±8.8 yr. During a mean follow up of 7.5 (±2.2) yr, 150 cases of lung cancer occurred. Using 7 h of sleep as the reference group, multivariable adjusted hazard ratios (95%CI) for lung cancer were 1.18 (0.77-1.82), 1.0 (ref), and 0.97 (0.67-1.41) from lowest to the highest category of sleep duration (*P* for quadratic trend 0.697), respectively. In a secondary analysis, smoking status did not modify the sleep duration-lung cancer association (*P*=0.78). Tere was no evidence for an interaction between sleep duration and sleep apnea on the risk of lung cancer either (*P*=0.65).

**Conclusions:**

Our data failed to show a higher risk of lung cancer in association with altered sleep duration among US male physicians.

## Introduction

Lung cancer is the most common cancer and cancer related cause of death worldwide^[1]^. Lung cancer has also been the leading cause of cancer related death in United States of America (USA)^[2]^. As per an estimate, there will be approximately 228, 190 individuals diagnosed with, and 159, 480 individuals dying of lung cancer in 2013^[3]^. Data from prior studies have demonstrated positive associations with genetic predisposition^[4, 5]^, occupational exposures^[6]^, inf lammation^[7, 8]^, type 2 diabetes (T2D)^[9, 10]^, a lcohol consumption^[4, 11]^, and most importantly smoking^[12]^ while beneficial effects of dietary factors^[13, 14]^, obesity^[15]^, and medium or high levels of physical activity^[16]^ on lung cancer risk have been reported. Among occupational exposures associated w ith increased risk of lung cancer include those involved with production of industrial machinery, metallurgical industry, ceramics industry, textile industry, rubber industry, and plastic industry among others^[6]^.

Others studies have demonstrated an increased risk of cancer and cancer related mortality in association with sleep disordered breathing (SDB)^[17, 18]^. A lterations in circadian rhythm has been associated with various aspects of carcinogenesis^[19, 20]^. Recently, study on a prospective cohort of Nurses' Health Study demonstrated a higher incidence of lung cancer with extended night-shift work among smokers^[21]^. In another study, a higher incidence of various cancers, including lung cancer was demonstrated among men who ever worked at night^[22]^. A lterations in sleep duration has also been associated with smoking^[23]^, alcohol consumption^[24]^, physical activity^[25]^, obesity^[26]^, inf lammation^[27, 28]^, T2D^[29]^, SDB^[30]^, and cancer related mortality^[31]^.

However, to the best of our knowledge, the association between sleep duration and incident lung cancer has not been investigated in a prospective cohort study. Therefore, the current study sought to prospectively assess the association of sleep duration with the risk of lung cancer among US male physicians.

## Methods

The Physicians' Health Study (PHS) Ⅰ is a completed randomized, double-blind, placebo-controlled trial, designed to study the effects of low-dose aspirin (ASA) and betacarotene on cardiovascular disease (CVD) and cancer among US male physicians. In 1997, PHS Ⅱ trial enrolled 7, 641 physicians from PHS Ⅰ along with 7, 000 new physicians to study the effects of vitamins on CVD and cancer. A detailed description of the PHS Ⅰ and Ⅱ has been published^[32, 33]^. Selfreported sleep duration was ascertained during the 2002 annual follow-up questionnaire which served as the baseline for our study. Of the total 29, 067 PHS subjects, we excluded people with prevalent lung cancer (*n*=63), and those who died before sleep duration assessment or had no followup (*n*=845), and those with missing data on sleep duration (*n*=7, 133). Thus, a final sample of 21, 026 participants was used for current analyses. Each participant gave written informed consent and the Institutional Review Board at Brigham and Women's Hospital approved the study protocol.

For self-reported sleep duration, participants were asked: "On average, how many total hours of sleep do you get in typical 24-h period?" Possible responses were " < 5 h", "6 h", "7 h", "8 h", "9 h", "10 h", and "11+h".

Incident cancer was ascertained through yearly followup questionnaires. Participants were asked: Since you flled out the last questionnaire (about 12 mo ago), were you newly diagnosed as having any of the following conditions? (Please mark No or Yes). One of the possible responses was, "Cancer (Non-skin). Site_____. Date of Diagnosis". All cancer cases were assessed and validated by medical record review by the PHS Ⅱ Endpoints Committee composed of physicians blinded to treatment assignment; 96.9% of confirmed total cancers were based on pathology or cytology reports. Cases of cancer were otherwise confirmed based upon strong clinical and radiological or laboratory marker evidence. Only confrmed cancer cases are included in this report.

Data on demographics, anthropometrics, exercise frequency, smoking, and alcohol consumption, along with history of T2D, sleep apnea, and snoring were obtained at baseline (*i.e*. 2002). If the data was not available at the baseline than the information from as close to sleep assessment as possible (usually from 12 mo prior questionnaire) was obtained. Age and body mass index (BMI) were used as continuous variables. Race was dichotomized as white *vs* non-white. Exercise was classifed as rarely/never, 1-3/mo, 1-4/wk, and 5-7/wk. Smoking was classified as never, past, and current smokers. For alcohol consumption, subjects were asked the following question: "How ofen do you usually consume alcoholic beverages?" Possible responses were: rarely/never, 1-3 times/mo, 1 time/wk, 2-4 times/wk, 5-6 times/wk, daily, and ≥2 times/d. These responses were interpreted as the number of alcoholic drinks consumed during the specifed period. For current analyses, alcohol consumption was classifed as < 1, 1-4, 5-7, and >7 drinks/wk. Diagnosis of T2D was selfreported and validated by detailed review of the medical records in a subsample^[34]^. Diagnosis of sleep apnea (Yes *vs* No) and snoring (rarely/never, few/occasionally, mostly/always, and unknown/missing), parental history of cancer, and caloric intake were based on self-reported information. For categorical variables, indicator values were created for missing observations.

We classified each subject into one of the following categories of average sleep duration: ≤6 h, 7 h, and ≥8 h. We computed person-time of follow up from the time when sleep duration was assessed until the first occurrence of a) confirmed lung cancer, b) death, or c) the date of last available follow up (August 1^st^, 2011). Baseline demographic variables were recorded and compared across categories of sleep duration.

We used *Cox* proportional hazard models to compute multivariable adjusted hazard ratios (HR) with corresponding 95% confidence intervals (CI) using participants reporting 7 h of sleep duration as the reference group. Potential confounding was assessed for established risk factors of lung cancer. First, we adjusted for age and race in model 1. Second, we also controlled for parental history of cancer, exercise frequency, caloric intake, BMI, T2D, alcohol consumption, smoking status, sleep apnea, and snoring in model 2.

To further address confounding by age we calculated agestandardized incidence rate for lung cancer using year 2000 US Standard Population. In secondary analyses, we evaluated whether there were statistically significant interactions between sleep duration and smoking status or sleep apnea by using a product term of both variables in a hierarchical model. Assumptions for proportional hazard models were tested (by including main effects and product terms of sleep duration and logarithmic-transformed time factor) and were met for all variables except snoring (*P* > 0.05). Therefore, snoring was included in a strata statement in the model. All analyses were conducted using SAS, version 9.2 (SAS Institute, NC). Signifcance level was set at 0.05 (two-sided test).

## Results

Table 1 shows baseline characteristics according to sleep duration. Mean age of the study participants at baseline was 68.3±8.8 yr. Long (≥8 h) sleep duration was associated with a lower prevalence of never smokers and frequent snoring; and a higher prevalence of elderly, white, >7/wk alcohol consumption, T2D, and higher caloric intake.

**1:** Baseline characteristic of 21, 026 United States male physicians according to average sleep duration

Variables	Sleep duration (h)			*P* value
	≤6.0 (*n*=5, 265)	7.0 (*n*=8, 606)	≥8.0 (*n*=7, 155)	
Age (mean±SD)	65.9±8.1	67.1±8.4	71.6±8.7	﹤0.000, 1
Body mass index (mean±SD)	26.2±3.7	25.8±3.4	25.7±3.6	0.142, 2
Race (% White)	4, 528 (86.5)	7, 878 (92.0)	6, 692 (94.0)	﹤0.000, 1
Smoking (%)				
Never	3, 049 (58.0)	4, 955 (57.6)	3, 634 (50.8)	﹤0.000, 1
Past	2, 067 (39.3)	3, 441 (40.1)	3, 269 (45.8)	﹤0.000, 1
Current	141 (2.7)	194(2.3)	243 (3.4)	0.005, 7
Exercise frequency (%)				
Rarely/never	1, 858 (35.8)	2, 749 (32.2)	2, 665 (37.5)	0.003, 6
1-3/mo	156(3.0)	218(2.6)	130(1.8)	﹤0.000, 1
1-4/wk	2, 251 (43.4)	4, 045 (47.4)	2, 986 (47.4)	0.070, 7
5-7/wk	922 (17.8)	1, 517 (17.8)	1, 328 (17.8)	0.112, 4
Alcohol consumption (drinks/wk) (%)				
﹤1/wk	1, 741 (33.3)	2, 440 (28.5)	2, 054 (28.8)	﹤0.000, 1
1-4/wk	1, 614 (30.8)	2, 489 (29.0)	1, 681 (23.6)	﹤0.000, 1
5-7/wk	1, 117 (21.3)	2, 151 (25.1)	1, 700 (23.8)	0.003, 7
>7/wk	764 (14.6)	1, 487 (17.4)	1, 696 (23.8)	﹤0.000, 1
History of snoring (%)				
Rarely/never	963(18.3)	1, 666 (19.3)	1, 481 (20.7)	0.000, 7
Few/occasionally	1, 984 (37.7)	3, 499 (40.7)	2, 836 (39.6)	0.054, 2
Mostly/always	1, 871 (35.5)	2, 743 (31.9)	2, 185 (30.6)	﹤0.000, 1
Unknown/missing	447 (8.5)	698 (8.1)	653 (9.1)	0.151, 6
Sleep apnea (%)	259 (4.9)	311 (3.6)	325 (4.6)	0.510, 4
Prevalent diabetes (%)	417 (7.9)	646 (7.5)	758 (10.6)	﹤0.000, 1
Parental history of cancer (%)	2, 499 (53.7)	4, 249 (54.8)	3, 338 (52.7)	0.189, 3
Calories (median, IQR)	1, 563 (1, 260-1, 951)	1, 612 (1, 314-1, 970)	1, 652 (1, 344-2, 034)	﹤0.000, 1

Table 2 shows a comparison of the baseline characteristics between subjects with missing data on sleep duration (afer excluding those who died before the assessment of baseline information and those with prevalent lung cancer) and those with complete data on sleep duration. Overall, there was higher prevalence of lung cancer among those with missing sleep duration than otherwise (293 *vs* 150 cases, respectively). The participants with missing data on sleep duration were more likely to be older; current smokers, heavy alcohol drinkers ( > 7/wk), had a higher prevalence of sleep apnea, and sedentary lifestyle. Tey however were less likely to have parental history of cancer and had a lower caloric intake compared to people with complete data on sleep duration.

**2:** Comparison of baseline characteristics between those with missing and people with complete data on sleep duration

Variables	Availability of data on sleep duration		*P* value
	No (*n*=7, 133)	Yes (*n*=21, 026)	
Age (mean±SD)	70.4±9.3	68.3±8.8	﹤0.000, 1
Body mass index (mean±SD)	25.3±3.8	25.9±3.5	﹤0.000, 1
Race (% White)	3, 945 (89.7)	19, 098 (91.3)	0.001
Smoking (%)			﹤0.000, 1
Never	2, 053 (46.6)	11, 638 (55.4)	
Past	2, 150 (48.7)	8, 777 (41.8)	
Current	207 (4.7)	578 (2.8)	
Exercise frequency (%)			﹤0.000, 1
Rarely/never	1, 880 (44.2)	7, 272 (34.9)	
1-3/mo	102 (2.4)	504 (2.4)	
1-4/wk	1, 568 (36.9)	9, 282 (44.6)	
5-7/wk	702 (16.5)	3, 767 (18.1)	
Alcohol consumption (drinks/wk) (%)			﹤0.000, 1
﹤1/wk	1, 450 (34.4)	6, 235 (29.8)	
1-4/wk	1, 101 (26.1)	5, 784 (27.6)	
5-7/wk	935 (22.1)	4, 968 (23.7)	
> 7/wk	732 (17.4)	3, 947 (18.9)	
History of snoring (%)			﹤0.000, 1
Rarely/never	84 (1.2)	4, 110 (19.5)	
Few/occasionally	173 (2.4)	8, 319 (39.6)	
Mostly/always	143 (2.0)	6, 799 (32.3)	
Unknown/missing	6, 733 (94.4)	1, 798 (8.6)	
Sleep apnea (%)	85 (5.8)	895 (4.3	0.007
Prevalent diabetes (%)	784(11.0)	1, 821 (8.7)	﹤0.000, 1
Parental history of cancer (%)	1, 019 (51.36)	10, 086 (53.84)	0.036
Calories (median, IQR)	1, 588 (1, 261-1, 908)	1, 616 (1, 312-1, 987)	﹤0.000, 1

During a mean follow up of 7.5 (±2.2) yr, 150 cases of lung cancer were diagnosed. Crude incidence rates of lung cancer were 0.86, 0.80, and 1.21 cases/1, 000 person-years for people reporting an average sleep duration of ≤6 h, 7 h, and ≥8 h, respectively (Table 3). Using 7 h of sleep as the reference group, multivariable adjusted hazard ratios (95%CI) for lung cancer were 1.18 (0.77-1.82), 1.0 (ref), and 0.97 (0.67-1.41) from lowest to the highest category of sleep duration (P for quadratic trend 0.697), respectively (Table 3).

**3:** Hazard ratios (95%CI) for lung cancer according to average sleep duration in Physicians' Health Study

Sleep duration (h)	Cases/personyears	Crude incidence rate (per 1, 000 person-yrs)	Hazards ratio (95%CI)		
			Unadjusted	Model 1	Model 2
≤6.0	35/40, 582	0.86	1.08 (0.71-1.66)	1.25 (0.82-1.92)	1.18 (0.77-1.82)
7.0	53/66, 352	0.80	1.0	1.0	1.0
≥8.0	62/51, 345	1.21	1.52 (1.05-2.19)	1.11 (0.76-1.60)	0.97 (0.67-1.41)
*P* for quadratic trend			0.155	0.348	0.697
Model 1: Age (< 60, 60- < 65, 65- < 70, 70- < 75, 75+) and race (white/non-white). Model 2: Age (< 60, 60- < 65, 65- < 70, 70- < 75, 75+), race (white/non-white), parental history of cancer (yes/no), exercise frequency (Never/Rarely, 1-3/mo, 1-4/wk, 5-7/wk), caloric intake (tertiles), body mass index (continuous), type 2 diabetes (yes/no), alcohol consumption (< 1/wk, 1-4/ wk, 5-7/wk, > 7/wk), smoking status (never, past, current), sleep apnea (yes/no), and snoring (mostly/always, few/occasionally, rarely/never, unknown/missing), alcohol consumption (< 1/wk, 1-4/wk, 5-7/wk, > 7/wk), smoking status (never, past, current), sleep apnea (yes/no), and snoring (mostly/always, few/occasionally, rarely/never, unknown/missing).					

The incidence rates for lung cancer from lowest to highest category of sleep duration were 8.62, 7.99, and 12.08 cases per 10, 000 person years. Age-standardized incidence rates (95%CI) were 10.0 (6.4-13.6), 7.4 (5.3-9.6), and 8.3 (6.0-10.7), respectively. In a secondary analysis smoking status did not modify the sleep duration-lung cancer association (*P*=0.78). There was no evidence for an interaction between sleep duration and sleep apnea on the risk of lung cancer either (*P*=0.65).

## Discussion

Our findings do not support an association of incident lung cancer with self-reported sleep duration among US male physicians. In a secondary analysis, stratifcation by smoking status failed to show an association between sleep duration and incidence of lung cancer. To the best of our knowledge, this is the first prospective study to assess the association between sleep duration and incident lung cancer.

There has been no prior study looking into the direct association between sleep duration and risk of lung cancer. However, there have been some studies providing an indirect support for an association between sleep duration and risk of lung cancer. Schernhammer *et al*^[21]^, in a prospective cohort of Nurses' Health Study demonstrated an increased risk of lung cancer among women who spent ≥15 yr working rotating night shifts [relative risk (RR)=1.28 (95%CI: 1.07-1.53)]. In an additional analysis the increased risk associated with ≥15 years of rotating night-shif work was limited to current smokers [RR=1.61 (95%CI: 1.21-2.13)].

Parent *et al*^[22]^, in a prior case-control study demonstrated a higher incidence of various cancers, including lung cancer [odd ratio (OR)=1.76 (95%CI: 1.25-2.47)], among men who ever worked at night compared to those who never did. However, they failed to show an increased risk of cancer with increased duration of night work. Also, the risk for lung cancer was not signifcant anymore for those who have been engaged in night work for more than 10 yr.

Despite a lack of association between sleep duration and incident lung cancer, alteration in sleep duration has been associated with factors considered important for developing lung cancer. Melatonin, secreted by pineal gland plays an important role in the regulation of circadian rhythm^[35]^. A lower level of melatonin has been associated with short sleep duration^[36]^. It has been demonstrated to act as a direct free radical scavenger, an indirect antioxidant, and as an important immunomodulatory agent^[37, 38]^. In a prior cross sectional study among human patients, a lower level of melatonin was demonstrated among those with non small cell lung cancer^[39]^.

In another study ﬂatening of diurnal cortisol rhythm, an indicator of abnormal circadian rhythm was demonstrated to be associated with worse prognosis among lung cancer patients^[40]^. Impairment in natural killer cell function, which play a critical role in cancer immunosurveillance, has also been associated with chronic circadian disruption in animal studies^[41]^.

Our study has some limitations. Information on average hours of sleep duration was self-reported and was assessed just once during the study period. There is no data on the quality of sleep. We do not know if reported sleep duration represented time spent in bed or actual sleep duration. We did not have information on the pathologic sub-types of lung cancer. Furthermore, participants in this study were male and mostly Caucasian physicians, thereby limiting the generalizability of our findings to general population. Nevertheless, the large sample size, more than 5 years of follow up, a standardized and systematic collection of covariates, availability of a large number of covariates, and a validation of lung cancer cases and comorbidities in the PHS are strengths of this study.

## Conclusion

In conclusion, our study failed to show a higher risk of lung cancer in association with altered sleep duration among US male physicians.

## Acknowledgements

We are indebted to the participants in the PHS for their outstanding commitment and cooperation and to the entire PHS staff for their expert and unfailing assistance.

The Physicians' Health Study is supported by grants CA-34944, CA-40360 and CA-097193 from the National Cancer Institute and grants HL-26490, and HL-34595, from the National Heart, Lung and Blood Institute of Health, Bethesda, MD. Funding agencies play no role in the data collection, ana lysis and manuscript preparation.
